# Mechanism of exosomal miR-155 derived from bone marrow mesenchymal stem cells on stemness maintenance and drug resistance in myeloma cells

**DOI:** 10.1186/s13018-021-02793-9

**Published:** 2021-10-24

**Authors:** Xinyu Gao, Jin Zhou, Jinghua Wang, Xiushuai Dong, Yuying Chang, Yinglan Jin

**Affiliations:** 1 Department of Hematology, The First Affiliated Hospital of Harbin Medical University, Harbin, Heilongjiang China; 2 Department of Hematology, The Second Affiliated Hospital of Harbin Medical University, Harbin, Heilongjiang China

**Keywords:** Multiple myeloma, Exosomes, miR-155, Myeloma cells, Stemness maintenance, Drug resistance

## Abstract

**Objective:**

This study was to explore the effect of exosomal miR-155 derived from bone marrow mesenchymal stem cells (BMSCs) on stemness maintenance and drug resistance in MPC-11 multiple myeloma cells.

**Methods:**

MPC-11 cells were transfected with mimics or inhibitors of miR-155. miR-155 expression was detected by qRT-PCR, cell condition was observed, and the expression of stemness maintenance markers OCT-4 and Nanog was observed by immunofluorescence. The expression of proteins associated with the Hedgehog signaling pathway and drug resistance was evaluated by western blot. To investigate whether exosomes affect cell behavior by horizontal delivery of miR-155, MPC-11 cells were co-cultured with exosomes isolated from BMSCs that were transfected with mimics or inhibitors of miR-155. Cell proliferation and the expression of proteins related to stemness maintenance protein and drug resistance were examined.

**Results:**

In function assays, after miR-155-mimics transfection, the expression levels of proteins related to stemness maintenance marker, Hedgehog signaling, and drug resistance were increased in MPC-11 cells. BMSC-derived exosomes carrying miR-155 inhibited apoptosis, promoted cell division, and upregulated the expression of protein associated with stemness maintenance, Hedgehog signaling, and drug resistance.

**Conclusion:**

Therefore, our findings indicate that exosomal delivery of miR-155 exerted the same effect as transfection did on the stemness maintenance and drug resistance of multiple myeloma cells.

**Supplementary Information:**

The online version contains supplementary material available at 10.1186/s13018-021-02793-9.

## Background

Multiple myeloma (MM) is a malignant tumor characterized by the proliferation of plasmacytes in the bone marrow microenvironment, and it is currently difficult to cure [1]. Immunomodulatory drugs such as bortezomib and thalidomide have anti-MM effects, indicating that maladjusted immune effector cells play an important role in MM [2–4]. Exosomes are vesicles with a diameter of 30–100 nm that are secreted by immune cells and cancer cells and exert immunomodulatory effects after external stimulation, participating in the formation of an immunosuppressive microenvironment in MM [5, 6]. In the tumor microenvironment, extracellular communication enables cells to coordinate and perform biological functions [7], and emerging evidence suggests that exosomes participate in tumor progression as a medium for cell communication [8]. In the progression of MM, exosomes regulate MM cells, mesenchymal stromal cells (MSC) and vascular endothelial cells (VEGF) in tumor microenvironment. (VEC), osteoclasts, immune cells and other cell interactions promote MM cell proliferation, angiogenesis, and the formation and differentiation of osteoclasts, which play an important role in MM immunosuppression [9]. Studies have suggested that secretion of exosome can act as a signal transduction medium to change the sensitivity of tumor to chemotherapy drugs and participate in tumor progress. Meanwhile, exosome can expel a variety of compound drugs out of tumor cells, reduce tumor cells’ drug resistance, and thus lead to the failure of chemotherapy [10]. miRNAs are small noncoding RNAs that can regulate gene expression in human cells. Specific miRNAs regulate cytokine expression and orchestrate proliferation and differentiation of stromal cell lines involved in the composition of the extracellular matrix [11, 12]. MiR-155 plays an important role in the drug resistance of various solid tumors such as glioma, non-small cell lung cancer, colorectal cancer, liver cancer, and breast cancer [13]. It also plays a crucial role in the occurrence and development of hematological tumors [14], but its specific underlying mechanism has not yet been clarified. Alexander et al. found that miR-155 regulated inflammation in an endotoxin-inflamed mouse model, emphasizing that miR-155 is carried by exosomes secreted by dendritic cells and can be absorbed by neighboring dendritic cells to promote inflammation [15] and confirming that miR-155 can be transported by exosomes between cells. In MM, whether miR-155 is involved in the drug resistance of myeloma cells has not been reported. We speculate that miR-155 is delivered by exosomes to affect the stemness maintenance of myeloma cells, thus affecting drug resistance. In this study, exosomes were isolated from mouse bone marrow mesenchymal stem cells (BMSCs), and the effect of miR-155 on myeloma cells (showing the same stem characteristics as BMSCs [16]) on stemness maintenance and drug resistance in mice was determined. The findings provide directions and references for clinically biological targeted therapy of MM.

## Methods

### Antibody and reagents

The following antibodies and reagents were purchased: Western blot used antibodies CD63 (Bioswamp, rabbit, PAB33929), CD81(abcam, rabbit, ab109201), TSG101 (Bioswamp, rabbit, PAB32949), Hhip (Bioswamp, rabbit, PAB40945), Ptch1 (Bioswamp, rabbit, PAB30041), Smo (Bioswamp, rabbit, PAB33618), GLi (Bioswamp, rabbit, PAB32098), GLiA (Bioswamp, rabbit, PAB30738), MRP1(Bioswamp, rabbit, PAB33537), ABCG2(Bioswamp, rabbit, PAB34152), P-gp (Bioswamp, rabbit, PAB30805), GAPDH(Bioswamp, rabbit, PAB36269), Goat anti-Rabbit IgG (Bioswamp, rabbit, PAB160011), flow cytometry used antibodies fluorescein isothiocyanate (FITC)-conjugated CD44 monoclonal antibody (eBioscience, 11-0441-82), allophycocyanin-conjugated CD29 (integrin beta 1) monoclonal antibody (eBioscience, 17-0291-82), FITC-conjugated CD11b monoclonal antibody (eBioscience, 11-0112-41), FITC-conjugated Ly-6A/E (Sca-1) monoclonal antibody (eBioscience, 11-5981-82), immunofluorescence used Nano antibodys (rabbit, Mitaka, 14295-1-AP), OCT-4 antibody (rabbit, Abcam, ab181557), Alexa Fluor 594-conjugated goat anti-rabbit antibody (Bioswamp, PAB160018), SYBR Green PCR Kit (KAPA Biosystems, KM4101), Lipofectamine® RNAiMAX (Invitrogen, 13778030), RPMI-1640 (Hyclone, SH30809.01B), chemiluminescence kit (Millipore, WBKLS0010), Lipofectamine® RNAiMAX (Invitrogen, 13778030), Trizol (Ambion, 15596026), and reverse transcription kit (TAKARA, 639505).

### Mice

The 4-week-old C57BL/6 mice (male) were from the Department of experimental animal, Harbin Medical University, weighing 16–18 g, and production license number SCXK (Hei) 2019-001. The study was approved by the Second Affiliated Hospital of Harbin Medical University Ethics Committee and adhered to the “Guidelines for Animal Care and Use of the Ethics Committee at Hospital of Harbin Medical University” (approval number sydw-2018-083).

### Mimics and inhibitors

The Cbfa1, OC, PPARγ2 et al. primers, miR-155-mimics and miR-155-inhibitors sequence were synthesized by Guangzhou Ruibo Biotechnology Co., Ltd.

### BMSC separation and identification

Male clean grade BALB/c mice (4–6 weeks) were housed at 22–26 °C in a 12-h light/dark cycle. According to the method of Yan et al. [17], the bilateral femurs were extracted on mice and the femoral bones were cut to expose the bone marrow cavity. The bone marrow cavity was repeatedly washed with the culture solution to collect and culture bone marrow cells. BMSCs were cultured in RPMI-1640 medium containing 15% fetal bovine serum (Gibco, 10270-106) and 1% Penicillin Streptomycin Solution (Solarbio, P1400) at 37 °C in an incubator with 5% CO_2_. Hematopoietic stem progenitor cells were isolated from the cells at passage 3 using CD11b magnetic beads. The expression of cell surface antigens CD11b, CD44, CD29, and SCA-1 was detected by flow cytometry.

### Exosome separation and identification and interaction with MPC-11 cells

The BMSCs were cultured in RPMI-1640 medium without FBS for 48 h, and cell culture medium was collected. The culture medium was centrifuged at 500×*g* for 10 min, and the supernatant was collected and centrifuged at 2000×*g* for 20 min. The supernatant was collected again and centrifuged at 100,000×*g* for 70 min. The precipitate was resuspended and centrifuged with a 40% sucrose gradient at 100,000×*g* for 70 min. The supernatant was collected and centrifuged at 100,000×*g* for 70 min, and the collected precipitate contained exosomes. Exosome was fixed with 2% glutaraldehyde (0.1 M PBS, pH7.4). Exosomes were isolated and observed under a transmission electron microscope. 1 × 10^6^ MPC-11 cells interact with 50 μg exosomes for 24 h. The expression of exosomal markers CD63, CD81, and TSG101 was detected using western blot [18].

### Exosome morphological identification of electron microscope

Exosome were fixed with 2% glutaraldehyde (0.1 M PBS, pH7.4) and integrated with nickel mesh and washing with PBS. The following is adding 1% glutaraldehyde dropwise to incubate for 5 min and washing with ddH2O several times. Next is adding 4% uranium acetate to the sample and incubated for 5 min. After drying, the exosome morphology was observed under the electron microscope.

### Cell transfection

The multiple myeloma cell line MPC-11 (Wuhan University Cell Bank, GDC300) and BMSCs were cultured in RPMI-1640 medium at 37 °C in an incubator with 5% CO_2_. 3 × 10^5^ cells in the logarithmic growth phase were seeded in 6-well plates with 2-mL cell culture medium. For transfection, 100 pmol of miR and 5 μL of Lipofectamine® RNAiMAX were mixed with 250 µL of Opti-MEM and added to 500 µL cell culture medium and then mixed with 1.5-mL cell culture medium. The cells were divided into five groups: control, miR-155-mimics, miR-155-inhibitors, miR-155-mimics-NC, and miR-155-inhibitor-NC.

### RNA extraction and quantitative reverse-transcription polymerase chain reaction (qRT-PCR)

Total RNA was extracted from cells using Trizol reagent, and cDNA was generated using the reverse transcription kit. qRT-PCR was performed on a CFX-Connect 96 instrument (Bio-Rad) using the SYBR Green Master Mix. The reaction mixture for qRT-PCR consisted of 10 μL of SYBR Green Master Mix, 1 μL of primer mix, 1 μL of cDNA template, and 8 μL of double distilled water. The conditions for qRT-PCR were as follows: pre-denaturation at 95 °C for 3 min; 40 cycles of denaturation at 95 °C for 5 s, annealing at 56 °C for 10 s, extension at 72 °C for 25 s; fluorescence signal acquisition between 65 and 95 °C. Using U6 as the internal reference gene, the primer sequences of each gene are shown in Table 1. 2^−ΔΔCt^ method was used to calculate the relative miR expression. The primers are listed in Table 1.

**Table 1:** Primer sequences for qRT-PCR

Primer	Sequence (5′–3′)
Cbfa1-F	GTGTTCTAGCCAAATCCT
Cbfa1-R	TTATGGGTGTTCCTCTGT
OC-F	GGGCAATAAGGTAGTGAA
OC-R	GTAGATGCGTTTGTAGGC
PPARγ2-F	GCAGAGCAAAGAGGTGGC
PPARγ2-R	TTTATTCATCAGGGAGGC
adipsin-F	AGAATGCCTCGTTGGGTC
adipsin-R	CGCAGATTGCAGGTTGTC
GAPDH-F	CCTTCCGTGTTCCTAC
GAPDH-R	GACAACCTGGTCCTCA
miR-155-RT-F	CTCAACTGGTGTCGTGGAGTCGG
miR-155-RT-R	CAATTCAGTTGAGACCCCTAT
miR-155-F	GGGTTAATGCTAATTGTG
miR-155-R	AACTGGTGTCGTGGAGTCGGC
U6-F	CTCGCTTCGGCAGCACA
U6-R	AACGCTTCACGAATTTGCGT

### Western blot

Cells were homogenized in protein lysate buffer, and debris was removed by centrifugation at 12,000×*g* for 10 min at 4 °C. After the addition of sample loading buffer, 50 μg of protein samples was electrophoresed and transferred to polyvinylidene difluoride membranes. The blots were blocked for 2 h at room temperature with fresh 5% nonfat milk in Tris-buffered saline/Tween 20 (TBST). The membranes were incubated with specific primary antibodies (CD63, CD81, TSG101, Hhip, Ptch1, Smo, Gli, GliA, MRP1, ABCG2, P-gp, GAPDH) in TBST overnight at 4 °C. After three washes with TBST, the blots were incubated with secondary antibodies (goat anti-Rabbit IgG) for 1 h, and the immunoreactive bands were visualized using an enhance chemiluminescence kit. The density of the immunoreactive bands was analyzed using TANON GIS software (TANON, China).

### Flow cytometric analysis of cell cycle

The collected 10^5^ cell suspension was centrifuged at 1000×*g* for 5 min, and the cell pellet was resuspended in 300 μL of phosphate-buffered saline (PBS) containing 10% fetal bovine serum. Absolute ethanol (700 µL) was added to fix the cells at -20 °C for at least 24 h. The fixed sample was centrifuged at 3000×*g* for 30 s, and the cell pellet was suspended in 100 μL of 1 mg/mL RNase A solution and incubated for 30 min at 37 °C. Propidium iodide (400 μL, 50 μg/mL) was added, and the nuclei were stained for 10 min in the dark. Flow cytometry was performed to determine the DNA content of the cells and the proportion of cells in each phase of the cell cycle. The results were analyzed using NovoExpress software (ACEA, China).

### Flow cytometry analysis of apoptosis

Ice-cold PBS (1 mL) was added to the collected cells, which were shaken gently and centrifuged at 4 °C at 1000×*g* for 5 min. The cells were resuspended in 200 μL of binding buffer. Then, 10 μL of annexin V-FITC and 10 μL of propidium iodide were added, mixed gently, and incubated at 4 °C in the dark for 30 min. Binding buffer (300 μL) was added, and flow cytometry was performed. The data were analyzed using NovoExpress software (ACEA, China).

### Immunofluorescence

The cells were fixed with 4% paraformaldehyde at room temperature for 30 min, washed with PBS, then permeabilized with 0.5% Triton X-100 at room temperature for 20 min. After three washes with PBS, the cells were blocked with 5% bovine serum albumin at 37 °C for 1 h, incubated with primary antibodies (Nanog, OTC-4) at room temperature for 1 h, and washed three times with PBS. Next, the cells were incubated with secondary antibody (Alexa Fluor 594—conjugated Goat Anti-Rabbit) at 37 °C for 1 h and washed three times with PBS. Cell nuclei were stained with DAPI for 20 min, and the cells were observed under a confocal laser scanning fluorescence microscope (Nikon, C2).

### Cell Counting Kit 8 (CCK8) assay

Cells in the logarithmic growth phase were seeded in 96-well plate at 5 × 10^3^ cells/well and transfected with mimics for 24 h. Then, 10 μL of CCK8 solution was added to each well and the cells were further incubated for 4 h. The absorbance of each well was measured at 450 nm using an enzyme-linked immunoassay detector (ALL FOR LIFE SCIENCE, AMR-100).

### Statistical analysis

All experiments were performed in triplicate (*n* = 3), and the data are expressed as means ± SE of the mean. All statistical analyses were performed using the GraphPad ProPrism 5.0 (San Diego, CA) software package. Student's t test and two-way analysis of variance were employed to analyze the differences between treatment groups. *P* < 0.05 was considered to be statistically significant.

## Result

### BMSC identification

**Fig. 1:**
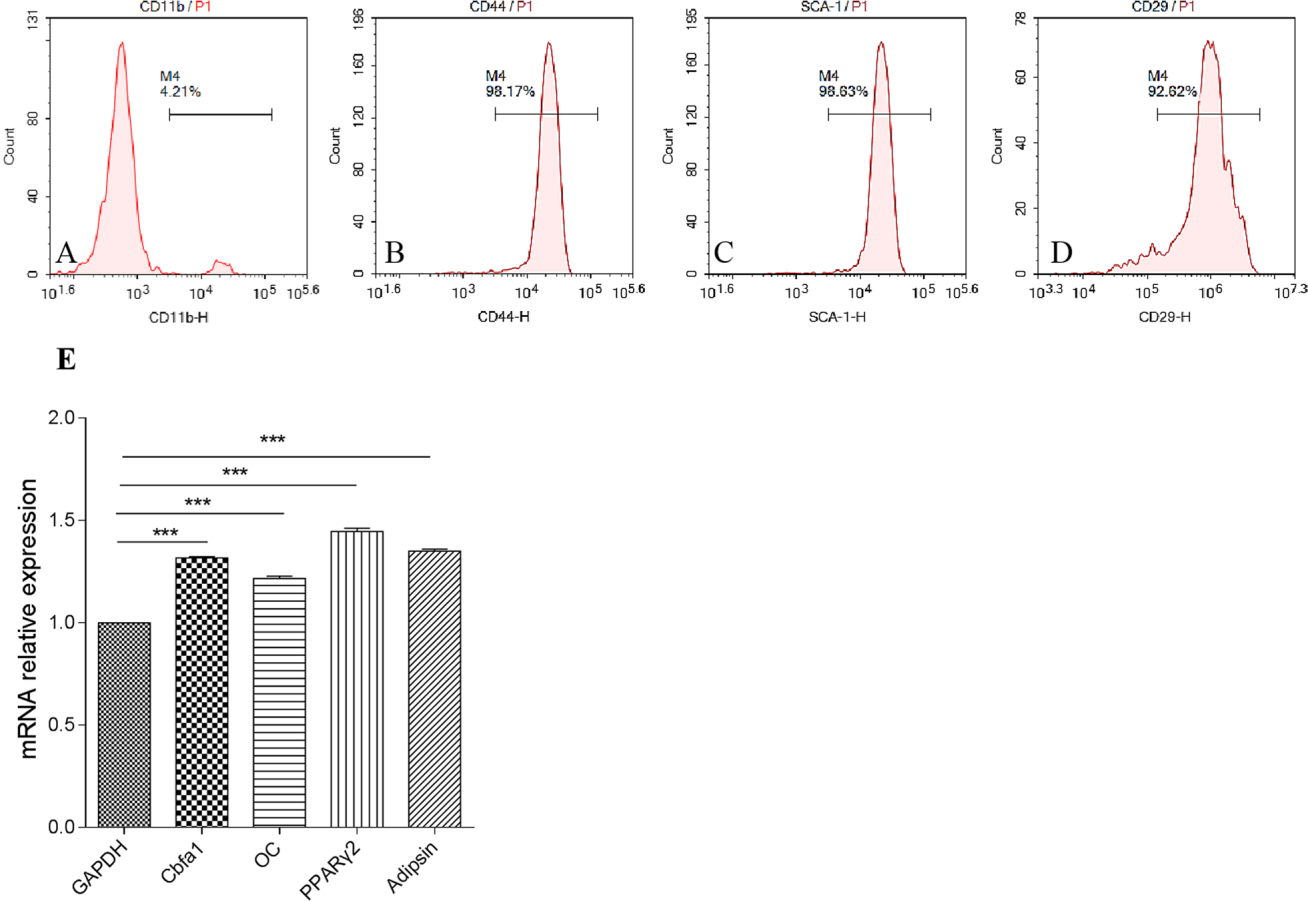
Proportion of **a** CD11b+, **b** CD44+, **c** SCA-1+, **d** CD29+ BMSCs, and **e** relative expression of Cbfa1, OC, PPARγ2, Adipsin

Hematopoietic stem progenitor cells with the surface marker CD11b account for a small proportion of BMSCs, and most BMSCs express CD44, SCA-1, and CD29. The proportion (Fig. 1) of CD44+, SCA-1+, and CD29+ cells was 98.17%, 98.63%, and 92.62%, respectively. In addition, BMSC osteogenic marker genes Cbfa1 and osteocalcin (OC) and adipogenic marker genes PPARγ2 and adipsin mRNA were detected by qRT-PCR, which result show the Cbfa1, OC, PPARγ2, adipsin abundantly expressed. These two results indicate that BMSCs was successfully separated.

### Exosome identification

**Fig. 2:**
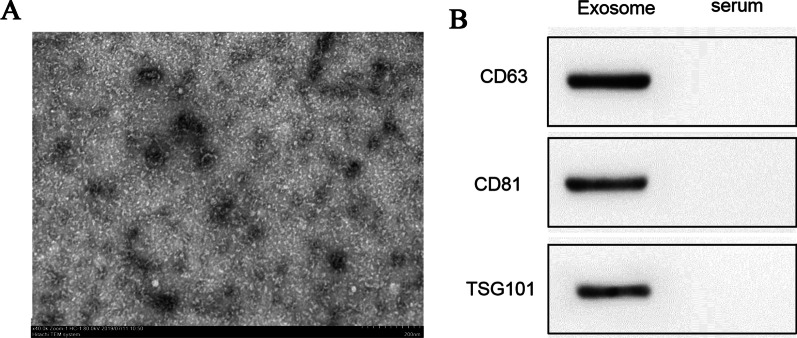
Exosome identification

The exosome markers CD63, CD81, and TSG101 were detected in the precipitate obtained by centrifugation as in Fig. 2, indicating that exosome separation was successful.

### MiR-155 detection

**Fig. 3:**
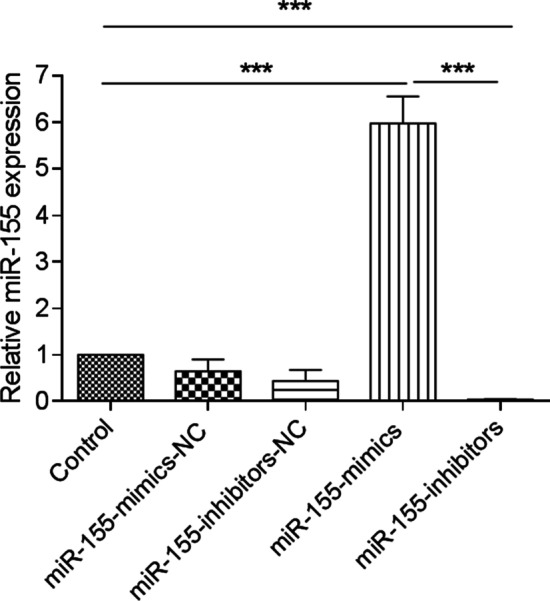
Relative expression of miR-155. ***P < 0.001

As shown in Fig. 3, 24 h after MPC-11 cells were subjected to transfection, miR-155 was abundantly expressed in the miR-155-mimics group and was significantly different from that in the control and miR-155-inhibitors group (*P* < 0.001). The miR-155 expression in the miR-155-inhibitor group was significantly lower than that in the control group (*P* < 0.001).

### Expression of proteins in Hedgehog signaling pathway after miR-155-mimics/inhibitors transfection

**Fig. 4:**
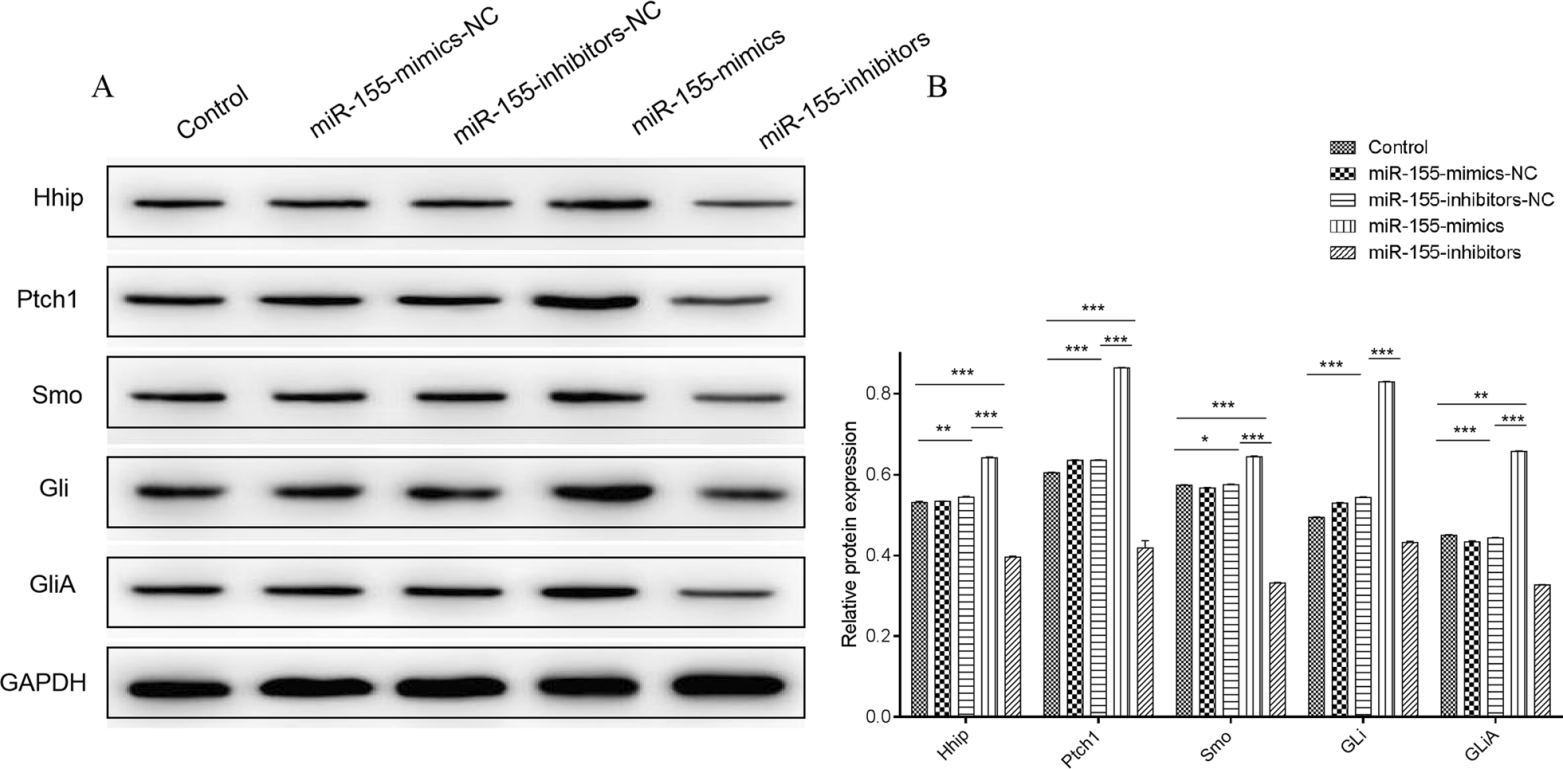
Protein expression of Hhip, Ptch1, Smo, Gli, and GliA after miR-155-mimics/inhibitors transfection for 24 h. **a** Western blot of proteins in the Hedgehog signaling pathway. **b** Quantification of relative protein expression. *P < 0.05; **P < 0.01; ***P < 0.001

Our findings indicate that exosomal delivery of miR-155 exerted the same effect as transfection did on the stemness maintenance and drug resistance of multiple myeloma cells (Additional file 1). After MPC-11 cells were transfected with miR-155-mimics/inhibitors for 24 h, the expression of proteins associated with the Hedgehog signaling pathway (Hhip, Ptch1, Smo, Gli, and GliA) was altered significantly. In Fig. 4, miR-155-mimics significantly increased the expression of these proteins compared to that of the control group (*P* < 0.05), whereas miR-155-inhibitors significantly reduced their expression compared with that of the control group (*P* < 0.05). There was no significant difference between miR-155-mimics-NC, miR-155-inhibitors-NC, and the control.

### Cell condition after miR-155-mimics/inhibitors transfection

**Fig. 5:**
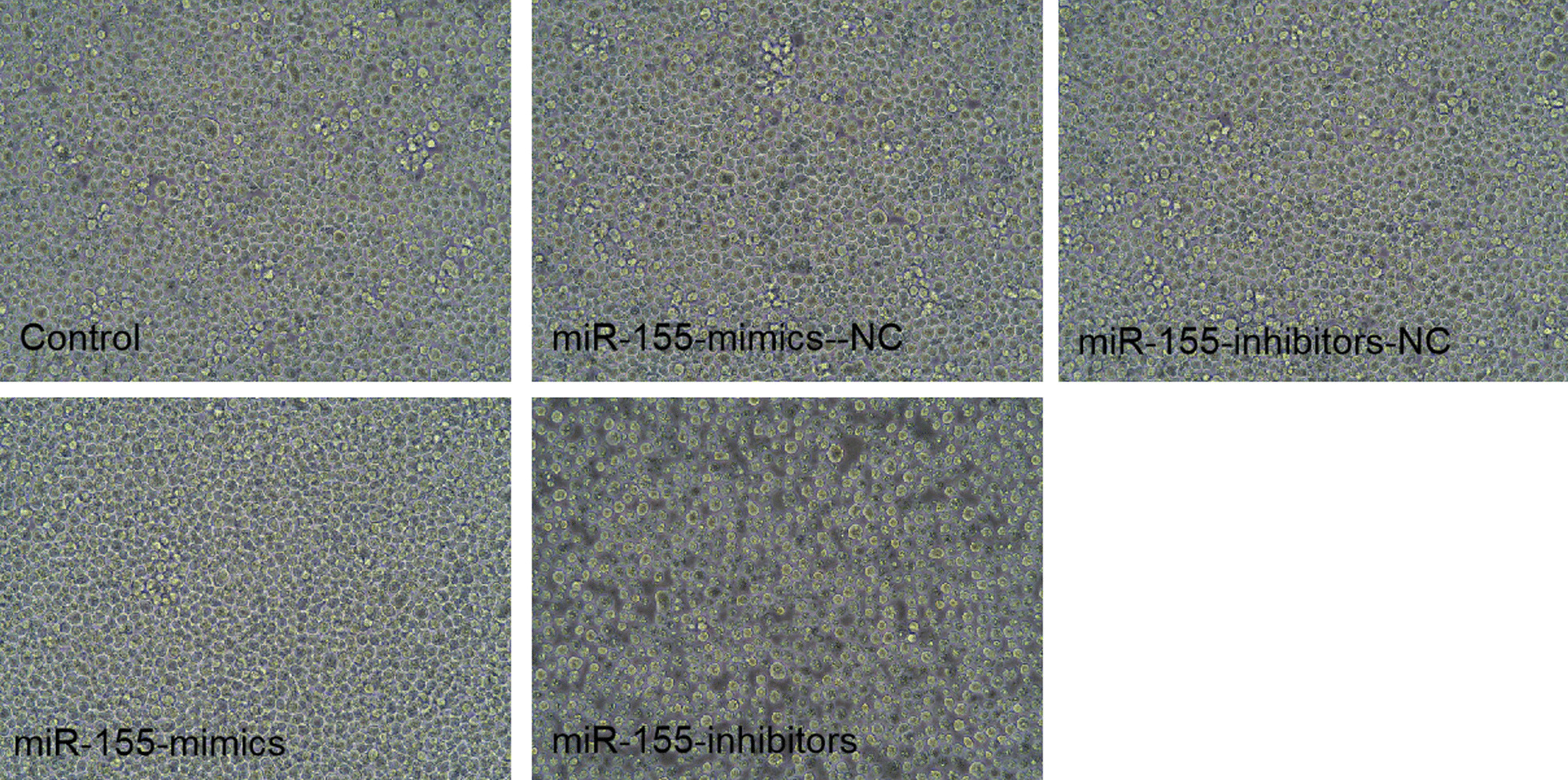
Cell condition after miR-155-mimics/inhibitors transfection for 24 h. Images were acquired at 200×

To confirm whether transfection of miR-155 was involved in cell viability, we transfected MPC-11 cells with miR-155-mimics and inhibitors. After incubated miR-155-mimics for 24 h, cell viability was increased compared with controls (Fig. 5). While the miR-155-inhibitors group were in poor condition, which presented cells atrophy and shed and terrible morphology. The cell condition in the NC group is similar to the control group.

### Stemness maintenance after miR-155-mimics/inhibitors transfection

**Fig. 6:**
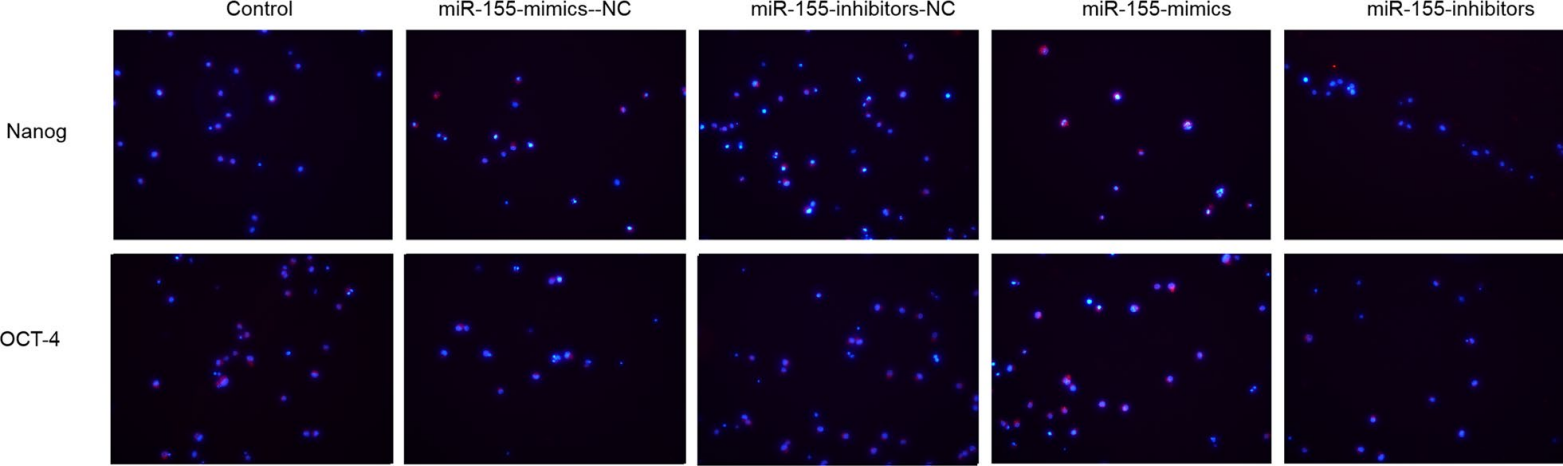
Expression of OCT-4 and Nanog after miR-155-mimics/inhibitors transfection for 24 h. Blue indicates the nucleus, red indicates Nanog and OCT-4 protein. Images were acquired at 200×

After 24 h of intervention, miR-155-mimics increased the expression of the stemness maintenance marker proteins OCT-4, and Nanog compared with that in the control group just like Fig. 6, whereas that in the miR-155-inhibitors group was reduced. No obvious differences were observed between the miR-155-mimics-NC, miR-155-inhibitors-NC, and control groups.

### Expression of drug resistance markers after miR-155-mimics/inhibitors transfection

**Fig. 7:**
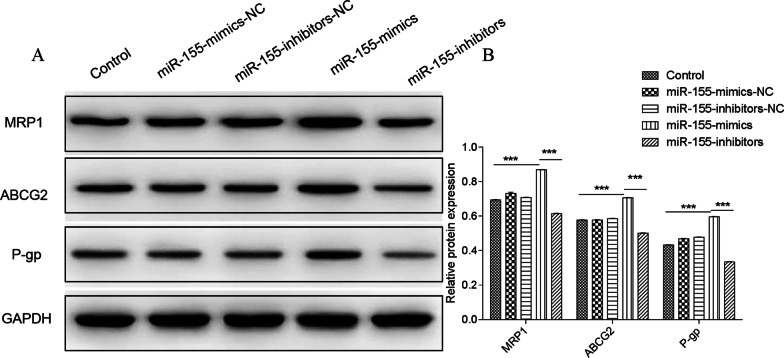
Protein expression of MRP1, ABCG2, and P-gp after 24 h of miR-155-mimics/inhibitors transfection. **a** Western blot of MRP1, ABCG2, and P-gp. **b** Quantification of relative protein expression. ***P < 0.001

As shown in Fig. 7, miR-155-mim significantly increased the expression of drug resistance-associated proteins MRP1, ABCG2 and P-gp compared with the control group 24 h after transfection (*P* < 0.001). However, miR-155-inhibitors significantly reduced the expression of these proteins (*P* < 0.001). No obvious differences were observed between the miR-155-mimics-NC, miR-155-inhibitors-NC, and control groups.

### Effect of BMSC-derived exosomes on MPC-11 cell apoptosis

**Fig. 8:**
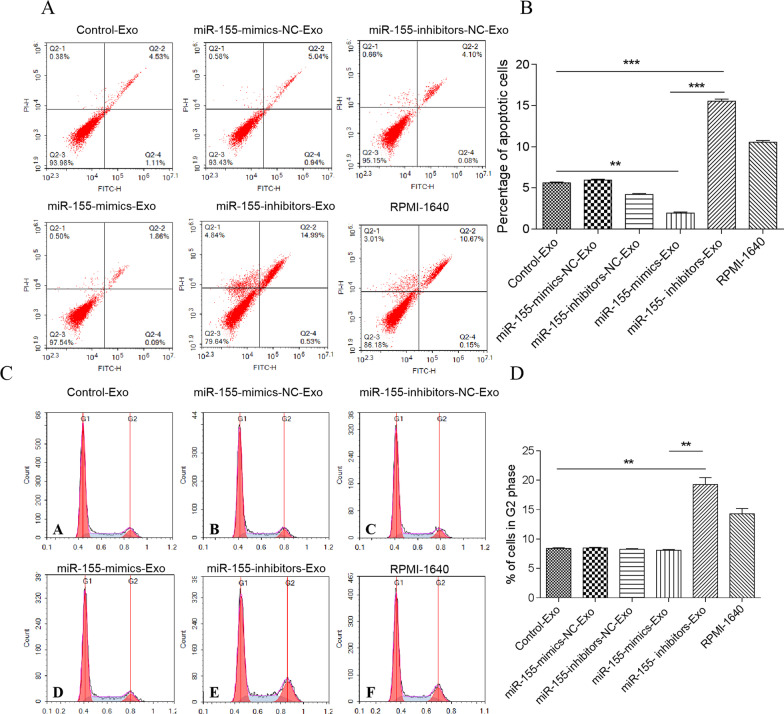
Flow cytometry of MPC-11 cells apoptosis and cell cycle after 24 h of co-culture with BMSC-derived exosomes. **a** Flow cytometry of cell apoptosis. The upper left quadrant represents necrotic cells, the lower left quadrant represents live cells, the upper right quadrant represents late apoptosis, and the lower right quadrant represents early apoptotic cells. **b** Percentage of apoptotic cells. **P < 0.01; ***P < 0.001. **c** Flow cytometry of cell cycle. **d** Percentage of cycle cells % of cells in G2 phase

MPC-11 cells were co-cultured with BMSC-derived exosomes for 24 h. In Fig. 8, the apoptosis of MPC-11 cells co-cultured with exosomes derived from miR-155-mimics (miR-155-mimics-Exo)-transfected BMSCs was significantly lower than that of MPC-11 cells co-cultured with exosomes from non-transfected (control) BMSCs (*P* < 0.01), whereas that of the miR-155-inhibitors-Exo group was significantly higher than that of Control-Exo (*P* < 0.001) and miR-155-mimics-Exo groups (*P* < 0.001). There was no significant difference between the NC and control groups.

miR-155-mimics-Exo and NC did not significantly affect cell cycle progression, whereas the proportion of cells in the G2 phase was significantly increased by miR-155-inhibitors-Exo compared to that in the control and miR-155-mimics-Exo groups (*P* < 0.01).

### Effect of BMSC-derived exosomes on MPC-11 cell condition

**Fig. 9:**
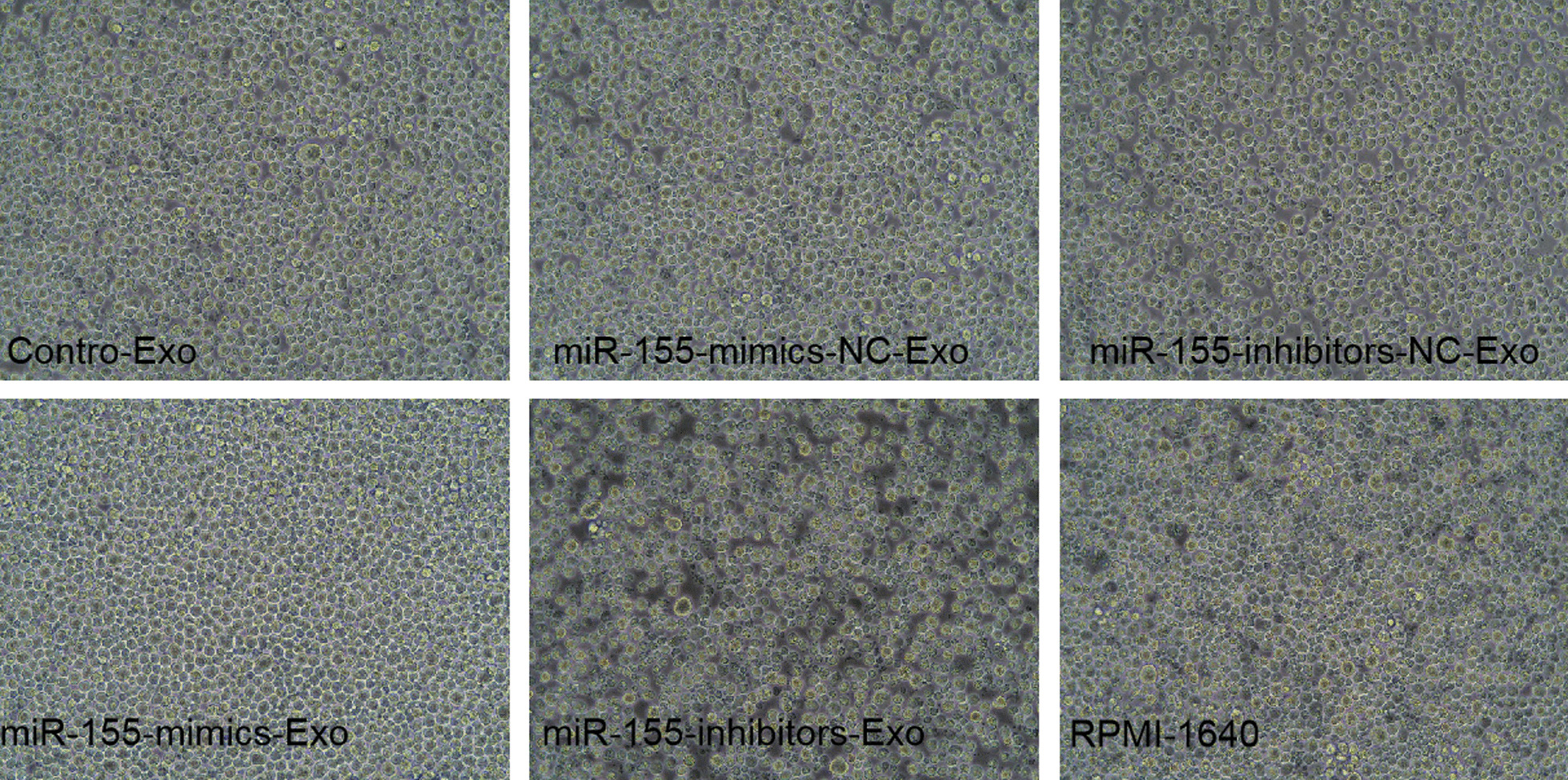
MPC-11 cell condition after 24 h of co-culture with BMSC-derived exosomes. Images were acquired at 200

After 24 h of co-culturing MPC-11 cells with BMSC-derived exosomes, the cell growth status of miR-155-mimics-Exo group was good as shown in Fig. 9, which presented favorable cell morphology and uniform growth, while miR-155-inhibitors-Exo group were in poor condition, which presented cells atrophy and shed and terrible morphology. The cell condition in the NC group is similar to the control group.

### Effect of BMSC-derived exosomes on stemness maintenance in MPC-11 cells

**Fig. 10:**
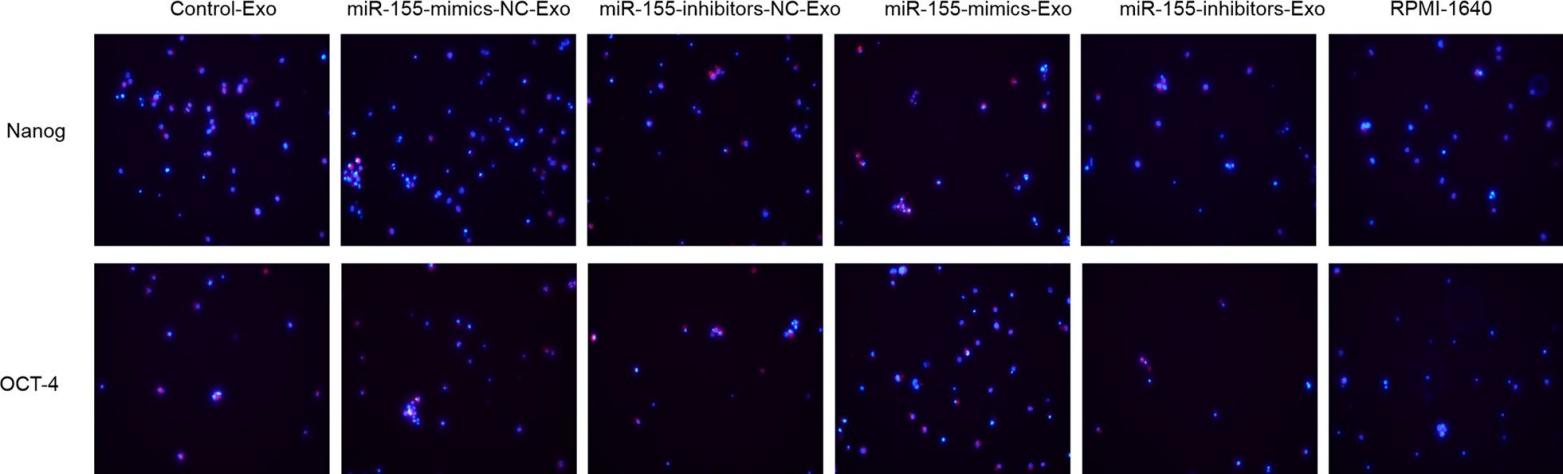
Expression of Nanog and OCT-4 after 24 h of co-culturing MPC-11 cells with BMSC-derived exosomes. Blue indicates the nucleus, red indicates Nanog and OCT-4 protein. Images were acquired at 200

After 24 h of co-culturing MPC-11 cells with BMSC-derived exosomes, the expression of Nanog and OCT-4 was increased by miR-155-mimics-Exo and decreased by miR-155-inhibitor-Exo compared to that in the control groups (Fig. 10).

### Effect of BMSC-derived exosomes on Hedgehog signaling pathway

**Fig. 11:**
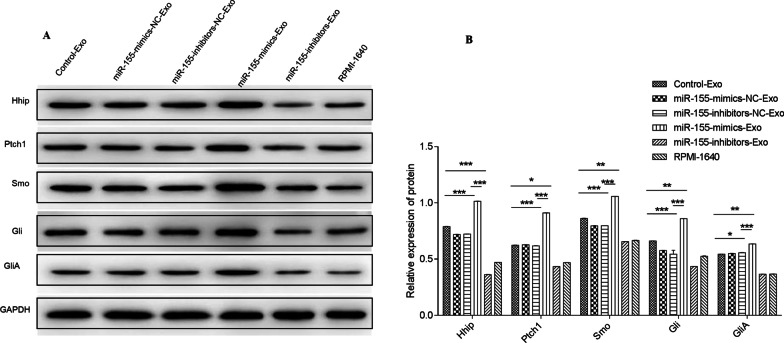
Protein expression of Hhip, Ptch1, Smo, Gli, and GliA after 24 h of co-culturing MPC-11 cells with BMSC-derived exosomes. *P < 0.05; **P < 0.05; ***P < 0.001

In Fig. 11, after 24 h of co-culturing MPC-11 cells with BMSC-derived exosomes, miR-155-mimics-Exo significantly upregulated the expression of proteins associated with the Hedgehog signaling pathway (Hhip, Ptch1, Smo, Gli, and GliA), which is related to stemness maintenance, compared to Control-Exo (*P* < 0.05). These proteins were significantly downregulated by miR-155-inhibitors-Exo (*P* < 0.05) compared to Control-Exo.

### Effect of BMSC-derived exosomes on MPC-11 cell proliferation

**Fig. 12:**
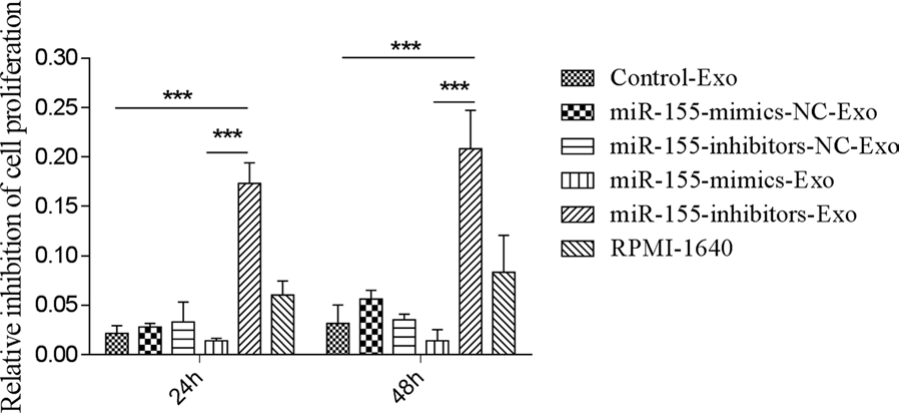
Inhibition of MPC-11 cell proliferation after 24 and 48 h of co-culturing MPC-11 cells with BMSC-derived exosomes. ***P < 0.001

After 24 h of co-culturing MPC-11 cells with BMSC-derived exosomes, the relative inhibition of MPC-11 cell proliferation induced by miR-155-mimics-Exo was lower than that induced by Control-Exo, but there was no significant difference (Fig. 12). However, miR-155-inhibitors-Exo induced significantly higher inhibition of MPC-11 cell proliferation than that induced by Control-Exo and miR-155-mimics-Exo (*P* < 0.001). The same trend was observed at 48 h.

## MiR-155-mimics promoted the expression of drug resistance-related proteins

**Fig. 13:**
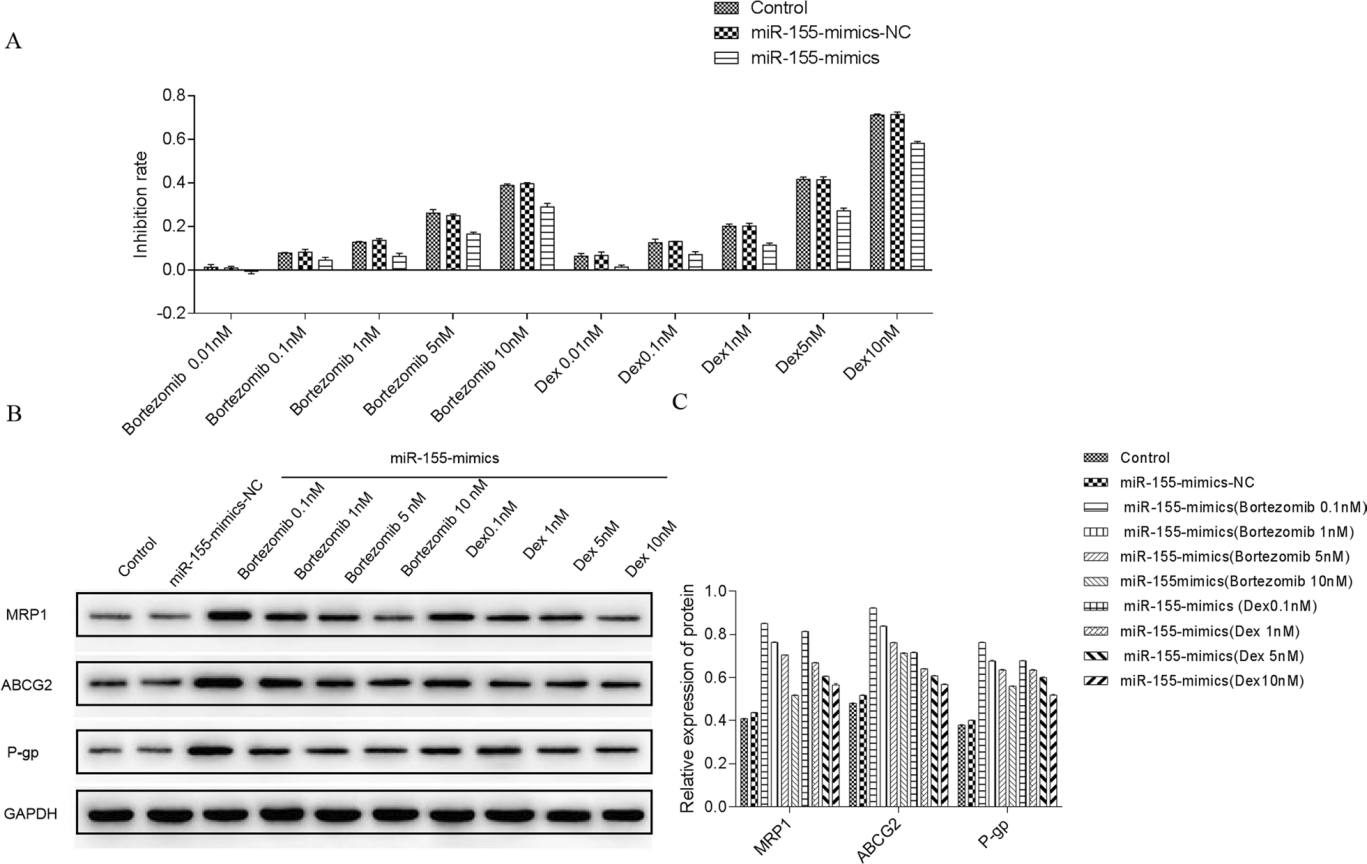
MiR-155-mimics promoted the expression of drug resistance-related proteins. Drug sensitivity of MPC-11 cells in each group was observed after 24 h. At the same time, the expressions of MRP1, ABCG2, and P-GP in all groups were observed with Western blot

After 24 h, the morphological changes before and after intervention were observed with 200 times light microscope. Bortezomib at different concentrations (0.01, 0.1, 1, 5, 10 nM) [1] and dexamethasone (0.01, 0.1, 1, 5, 10uM) were used to intervene in the miR-155-mimics group and the control group. Drug sensitivity of MPC-11 cells in each group was observed after 24 h. At the same time, the expressions of MRP1, ABCG2 and P-GP in all groups were observed with Western blot (Fig. 13).

## Discussion

MM is also known as plasma cell myeloma and accounts for 1% of all tumors and 13% of blood tumors. Genome sequencing and analysis found that genes involved in protein translation, including methylation genes and those involved in blood clotting, have undergone mutations in MM patients. Mutations in signaling pathways such as nuclear factor-κB were also discovered [19]. The genetic complexity of MM makes it difficult to target mutations or specific signaling pathways. Studies have reported that the incidence of MM is closely related to the bone marrow microenvironment [20], and the identification of biological targets from the cell or tumor microenvironment is an urgent issue. miR-155 participates in various physiological activities and has become a therapeutic target and biomarker in many tumors. Feng et al. reported that miR-155 promoted the proliferation, migration, and colony formation of MM cell lines (RPMI-8266 and U266) through in vitro interference experiments and demonstrated that miR-155 suppressed the expression of the tumor suppressor gene SOCS1, thereby regulating JAK/STAT signaling [21]. A recent study showed that miR-155 plays an important role in the development of drug resistance in a variety of solid tumors, but its specific mechanism in blood tumors, especially MM, has not been elucidated [13]. We transfected the MM cell line MPC-11 with mimics or inhibitors of miR-155 and found that after miR-155 was successfully expressed in MPC-11 cells after 24 h of miR-155-mimics transfection. The Hedgehog (Hh) signaling pathway plays a crucial role in maintaining the stemness of MM cells, as it can inhibit clonal expansion and promote the terminal differentiation of MM stem cells [22]. OCT-4 is a member of the POU domain transcription factor family that is usually expressed on pluripotent stem cells and plays a vital role in maintaining embryonic development and the proliferation and differentiation of stem cells. Nanog plays a momentous role in maintaining the self-renewal of cancer cells and the characteristics of cancer stem cells. Nanog and OCT-4 overexpression mediate the drug sensitivity and proliferation of various tumor cells [23]. After miR-155-mimics transfection, the expression of proteins involved in the Hedgehog signaling pathway, namely Hhip, Ptch1, Smo, Gli, and GliA (an activated form of Gli), was increased significantly. Moreover, OCT-4 and Nanog were significantly upregulated, indicating that miR-155 promoted stemness maintenance in MPC-11 cells. The drug resistance-related proteins MRP1, ABCG2, and P-gp play critical roles in drug sensitivity and confer drug resistance to cells. After miR-155-mimics transfection, the expression of MRP1, ABCG2, and P-gp increased significantly compared with that in control cells, indicating that miR-155 enhanced the drug resistance of MPC-11 cells. In the bone marrow microenvironment, MM cells interact with bone cells to enhance bone resorption activity and destroy new bone formation. In turn, BMSCs provide a framework for survival and drug resistance [24]. Peacock et al. found that exosomes are an important medium for crosstalk between MM cells and bone marrow microenvironment. Exosomes produced in the tumor microenvironment can interact with target cells through the following mechanisms: (1) direct stimulation of ligands expressed on the surface of the target cell membrane; (2) receptor transfer between the tumor and the target cell; (3) horizontal transfer of genetic information (miRNAs, mRNA, etc.) to target cells; (4) direct stimulation of ligands on the membrane surface of target cells through endocytosis [15, 25]. To investigate whether miR-155-mimics/inhibitors can affect MPC-11 cells by exosomal transfer, we isolated exosomes from BMSCs that were transfected with miR-155-mimics/inhibitors and co-cultured MPC-11 cells with the exosomes for 24 h. We observed that miR-155 was delivered by exosomes and promoted the expression of proteins in the Hedgehog signaling pathway as well as OCT-4 and Nanog, indicating that miR-155-mimics-Exo promoted stemness maintenance in MPC-11 cells. In addition, miR-155-mimics-Exo promoted the expression of drug resistance-related proteins MRP1, ABCG2, and P-gp, suggesting that miR-155 increased the drug resistance of MPC-11 cells. It is worth noting that miR-155-mimics-Exo induced the lowest number of apoptotic cells, indicating that miR-155 can inhibit apoptosis. In addition, miR-155-inhibitors induced the highest proportion of cells in the G2 phase, demonstrating that miR-155-mimics promoted cell division.

In summary, miR-155 promoted cell growth and stemness maintenance and enhanced the drug resistance of MPC-11 cells. In addition, miR-155 was horizontally delivered by exosomes to produce the same effect.

## Supplementary Information

**Additional file 1**. BMSC-derived exosomes carrying miR-155 inhibited apoptosis, promoted cell division, and upregulated the expression of protein associated with stemness maintenance, Hedgehog signaling, and drug resistance.

## Data Availability

The datasets used and/or analyzed during the current study are available from the corresponding author on reasonable request.

## Abbreviations

BMSCs: Bone marrow mesenchymal stem cells
MM: Multiple myeloma
FITC: Flow cytometry used antibodies Fluorescein isothiocyanate
TBST: Tris-buffered saline/Tween 20
PBS: Phosphate-buffered saline
OC: Osteocalcin
Hh: Hedgehog
