# Supplementary material for: Mechanism of exosomal miR-155 derived from bone marrow mesenchymal stem cells on stemness maintenance and drug resistance in myeloma cells
Source: J Orthop Surg Res. 2021 Oct 24;16:637. doi: 10.1186/s13018-021-02793-9 (PMC8543846; doi:10.1186/s13018-021-02793-9)
Supplement: Supplementary file 1 — Additional file 1. BMSC-derived exosomes carrying miR-155 inhibited apoptosis, promoted cell division, and upregulated the expression of protein associated with stemness maintenance, Hedgehog signaling, and drug resistance. [file 13018_2021_2793_MOESM1_ESM.docx]

Mechanism of exosomal miR-155 derived from bone marrow mesenchymal stem cells on stemness maintenance and drug resistance in myeloma cells

Xinyu Gao^a,b^, Jin Zhou^a*^, Jinghua Wang^b^,Xiushuai Dong^b^_,_ Yuying Changb, Yinglan Jin^b^

^a^Department of Hematology,The First Affiliated Hospital of Harbin Medical University, Harbin, Heilongjiang, China;^b^Department of Hematology,The Second Affiliated Hospital of Harbin Medical University, Harbin, Heilongjiang, China.


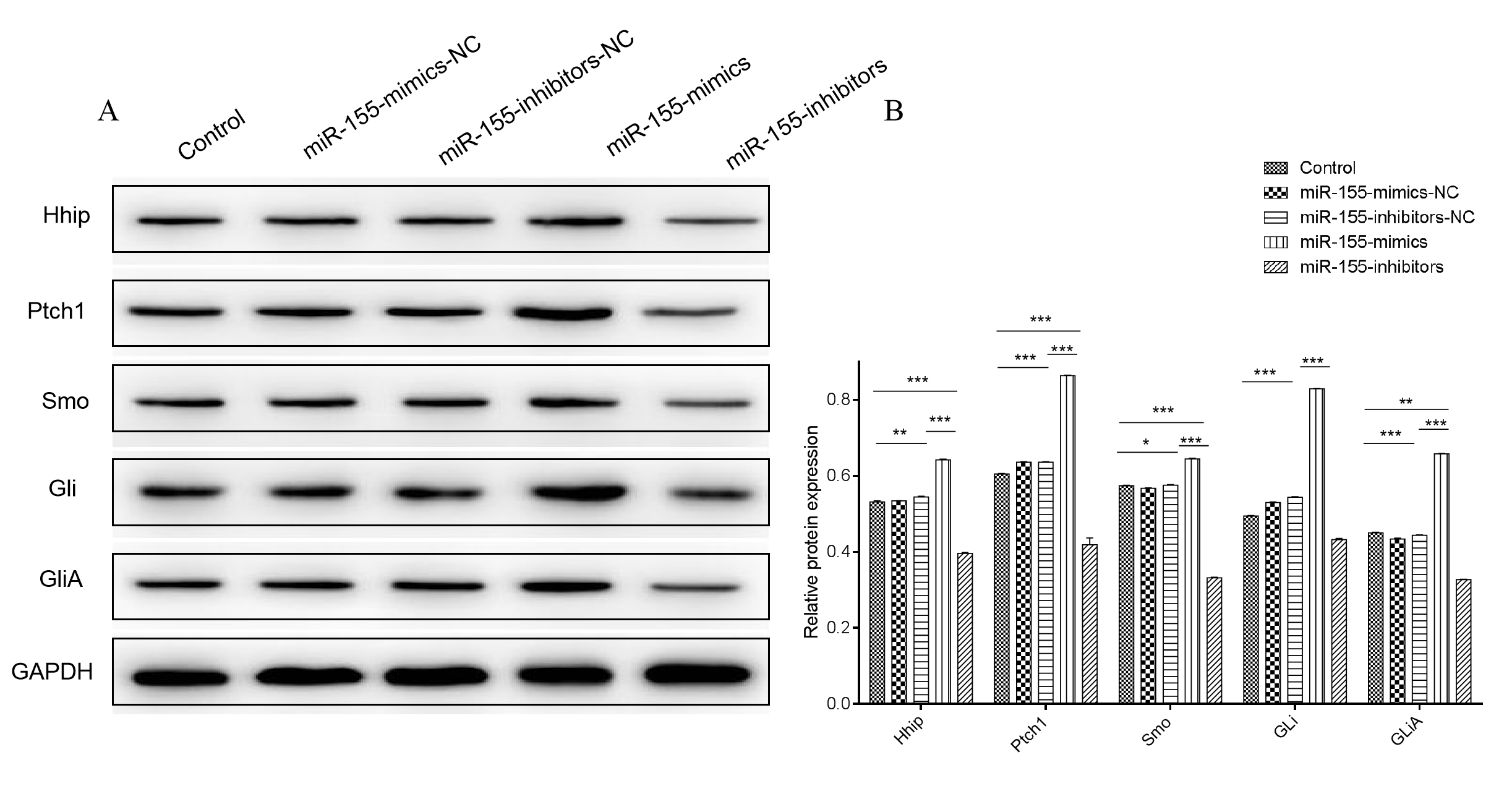


BMSC-derived exosomes carrying miR-155 inhibited apoptosis, promoted cell division, and upregulated the expression of protein associated with stemness maintenance , Hedgehog signaling, and drug resistance.

Our findings indicate that exosomal delivery of miR-155 exerted the same effect as transfection did on the stemness maintenance and drug resistance of multiple myeloma cells.
